# Supplementary material for: Analysis of PPARγ Signaling Activity in Psoriasis
Source: Int J Mol Sci. 2021 Aug 10;22(16):8603. doi: 10.3390/ijms22168603 (PMC8395241; doi:10.3390/ijms22168603)
Supplement: Supplementary file 1 [file ijms-22-08603-s001.zip › Supplemental materials_Analysis of PPARg signaling activity in psoriasis/Pathway models/Models images and html files/PPARG expressed in cells assotiated with psoriasis/225533.html]

monocyte


# CellType monocyte

|  |  |
| --- | --- |
| URN | urn:agi-ncimcelltype:C0026473 |
| Total Entities | 72 |
| Connectivity | 9599 |
| Name | monocyte |

---

|  |  |
| --- | --- |
| ChildConcepts | monocyte adhesion |
|  | monocyte aggregation |
|  | monocyte chemotaxis |
|  | monocyte differentiation |
|  | monocyte homeostasis |
|  | activated monocyte |
|  | classical monocyte |
|  | inflammatory monocyte |
|  | intermediate monocyte |
|  | non-classical monocyte |
|  | resident monocyte |
|  | monocyte like cell |
|  | lymphomonocyte |
|  | monocyte infiltration |
|  | monocyte function |
|  | monocyte migration |
|  | monocyte development |
|  | monocyte motion |
|  | monocyte transendothelial migration |
|  | monocyte contact |
|  | monocyte rolling |
|  | monocyte survival |
|  | monocyte proliferation |
|  | monocyte lifespan |
|  | monocyte growth |
|  | monocyte macrophage differentiation |
|  | monocyte population |
|  | monocyte apoptosis |
|  | monocyte motility |
|  | monocyte phenotype |
|  | monocyte polarity |
|  | monocyte formation |
|  | monocyte tethering |
|  | monocyte invasion |
|  | monocyte fate commitment |
|  | monocyte fate |
|  | monocyte extravasation |
|  | monocyte priming |
|  | monocyte damage |
|  | monocyte localization |
|  | monocyte spreading |
|  | monocyte death |
|  | monocyte homing |
|  | monocyte phagocytosis |
|  | monocyte mediated cytotoxicity |
|  | monocyte division |
|  | monocyte phagocyte activity |
|  | monocyte transdifferentiation |
|  | monocyte cytotoxicity |
|  | monocyte diapedesis |
|  | monocyte sequestration |
|  | monocyte distribution |
|  | monocyte repertoire |
|  | monocyte structure |
|  | monocyte renewal |
|  | monocyte recognition |
|  | monocyte stress |
|  | monocyte selection |
|  | monocyte destruction |
|  | monocyte autophagy |
|  | monocyte volume |
|  | monocyte ratio |
|  | monocyte size |
|  | monocyte transepithelial migration |
|  | monocyte clonal anergy |
|  | monocyte senescence |
|  | monocyte disruption |
|  | monocyte granulocyte differentiation |
|  | monocyte proliferative response |
|  | monocyte regeneration |
|  | monocyte fate specification |
|  | monocyte agglutination |

---

|  |  |
| --- | --- |
| Pathway | Elevated Receptors -> Expression Targets in Lymphoid System and Blood |
|  | CD Markers -> Expression Targets in Lymphoid System and Blood |
|  | GPCRs Family -> Expression Targets in Lymphoid System and Blood |
|  | PPARG expressed in cells assotiated with psoriasis |
|  | Neighbors of monocyte |
|  | 1\_Low-density lipoprotein internalization and the accumulation of lipids in atherosclerosis |

---

|  |  |
| --- | --- |
| MedScan ID | 10000095 |

---

|  |  |
| --- | --- |
| Alias | monocytic |
|  | monocytal |
|  | monocytes |
|  | monocyte |

---

|  |  |
| --- | --- |
| NCIm ID | C0026473 |

---
